# Combining electroacupuncture and transcutaneous electrical acupoint stimulation for psychiatric disorders in women victims of domestic violence: An assessor‐blinded, randomised controlled trial

**DOI:** 10.1002/gps3.70035

**Published:** 2026-07-07

**Authors:** Sichang Yang, Wai Chun Mok, Denise Shuk Ting Cheung, Agnes Tiwari, Calvin Pak Wing Cheng, Haiyong Chen, Zongshi Qin, Mei Yan Chan, Xiaoru Hu, Kam Chi Yu, Pui Yan Chan, Wenqi Li, Sheng‐Nan Liu, Min‐Qing Gu, Xinjing Yang, Doris Lee, Sai Ho Sin, Zhang‐Jin Zhang

**Affiliations:** ^1^ School of Chinese Medicine, LKS Faculty of Medicine The University of Hong Kong Hong Kong China; ^2^ Department of Chinese Medicine The University of Hong Kong‐Shenzhen Hospital (HKU‐SZH) Shenzhen Guangdong China; ^3^ School of Nursing, LKS Faculty of Medicine The University of Hong Kong Hong Kong China; ^4^ School of Nursing Hong Kong Sanatorium and Hospital Hong Kong China; ^5^ Department of Psychiatry, LKS Faculty of Medicine The University of Hong Kong Hong Kong China; ^6^ Peking University Clinical Research Institute Peking University Beijing China; ^7^ Department of Acupuncture and Moxibustion Shenzhen Hospital (Fu Tian) of Guangzhou University of Chinese Medicine Shenzhen Guangdong China; ^8^ Harmony House Ltd., Kowloon Hong Kong China; ^9^ The Hong Kong Buddhist Association ‐ The University of Hong Kong Chinese Medicine Clinic cum Training and Research Centre (Wong Tai Sin District) Hong Kong China

## Abstract

**Background:**

Women victims of domestic violence often present with psychiatric disorders and may benefit from nonpharmacological interventions. Acupoint stimulation therapy may be an effective approach.

**Aims:**

To examine whether a combination of electroacupuncture (EA) and transcutaneous electrical acupoint stimulation (TEAS) can alleviate depression, post‐traumatic stress disorder (PTSD) and insomnia in women victims of domestic violence.

**Methods:**

An assessor‐blinded randomised controlled trial was conducted in Hong Kong, China, with 110 Chinese women with major depressive disorder who had experienced domestic violence and were randomly assigned to care as usual (CAU) alone or combined with EA + TEAS (*n* = 55 per group) for 12 weeks, two clinic‐based EA sessions and three home‐based TEAS sessions per week. The primary outcome was baseline‐to‐endpoint change in the Beck Depression Inventory‐II (BDI‐II). Secondary outcomes included the 17‐item Hamilton Depression Rating Scale, 10‐item Perceived Stress Scale, PTSD Check List‐Civilian Version, Insomnia Severity Index and the 12‐item Short Form Survey for health‐related quality of life.

**Results:**

Of the 110 participants (*n* = 55 each group), 91.8% (101/110) completed the study. At 12 weeks, the EA + TEAS group showed a significantly greater reduction in BDI‐II score than that of the CAU group (mean difference = −10.9; 95% confidence interval −16.5 to −5.4; *t* = −3.9; *p* < 0.001), exceeding the minimal clinically important difference (5 points). The superiority of EA + TEAS was also observed across secondary measures, including depression, PTSD, perceived stress, insomnia and quality of life. The EA + TEAS group had significantly higher rates of clinical response (49.1% vs. 18.2%, *χ* ^2^ = 11.770, *p* < 0.001) and remission (38.2% vs. 12.7%, *χ* ^2^ = 9.390, *p* = 0.002) at endpoint than those of the CAU group. All treatment‐related adverse events were mild.

**Conclusions:**

The addition of EA + TEAS produced substantially greater improvements in depression and other psychiatric symptoms. EA + TEAS may serve as an effective intervention for women victims of domestic violence.

**Trial Registration:**

This trial was registered on www.clinicaltrials.gov (NCT05102253).

## Abstract (highlights)

**WHAT IS ALREADY KNOWN ON THIS TOPIC**

- Women victims of domestic violence often present with psychiatric disorders such as depression, post‐traumatic stress disorder and insomnia. However, targeted treatments for the psychiatric symptoms of this population remain lacking.

**WHAT THIS STUDY ADDS**

- This study demonstrates that adding a combination of electroacupuncture (EA) and transcutaneous electrical acupoint stimulation (TEAS) to care as usual is both effective and safe for alleviating psychiatric symptoms in women victims of domestic violence.

**HOW THIS STUDY MIGHT AFFECT RESEARCH, PRACTICE OR POLICY**

- The combination of EA and TEAS may serve as an effective adjunctive intervention for depression and other psychiatric symptoms in women victims of domestic violence, potentially informing clinical practice and guiding future research and policy development for this vulnerable population.

## INTRODUCTION

Domestic violence against women is a serious and pervasive public health issue worldwide. Globally, approximately one in three (30%) women have experienced physical and/or sexual violence in their lifetime.^1^ The prevalence of physical violence against women was 4.5%–10% in Hong Kong, China^2^, ^3^ and 16%–26% in China.^4^ Such exposure to violence results in a wide spectrum of negative psychological sequelae, in particular, depression, post‐traumatic stress disorder (PTSD) and sleep disturbance.^5^, ^6^, ^7^ Although advocacy and psychotherapy have served as first‐line interventions for victims of domestic violence, their effectiveness in alleviating post‐traumatic psychiatric disorders remains limited.^8^, ^9^ Moreover, as a vulnerable population, abused women often face numerous barriers to accessing healthcare. For instance, substantial time commitments and high costs have largely limited access to psychological and pharmacological therapy.^10^ Therefore, there is a pressing need for alternative therapeutic strategies that offer comparable efficacy while being highly accessible and acceptable.

Acupoint stimulation therapy (AST) refers to a group of techniques involving either invasive or noninvasive stimulation of acupoints.^11^ The two most commonly used forms of AST are electroacupuncture (EA), a clinic‐based procedure, and transcutaneous electrical acupoint stimulation (TEAS), which can be self‐administered at home. Owing to their high accessibility, acceptability and effectiveness, both modalities have been increasingly used to treat a range of psychiatric disorders. A large body of evidence suggests the efficacy of EA in treating depression,^12^, ^13^ PTSD^14^ and insomnia.^15^, ^16^ We developed a novel EA mode called dense cranial electroacupuncture stimulation (DCEAS), which delivers EA to forehead acupoints innervated by the trigeminal nerve.^13^ The efficacy of DCEAS has been demonstrated in patients with major depression,^13^ obsessive–compulsive disorder (OCD),^17^ post‐stroke depression^18^ and chemotherapy‐induced cognitive impairment.^19^ Beyond its established efficacy for postoperative pain and sleep problems,^20^, ^21^, ^22^ our studies have demonstrated that TEAS is also effective as monotherapy and adjunctive therapy for mild‐to‐moderate depression, PTSD and OCD.^23^, ^24^, ^25^ Notably, individuals with depression and a history of psychological trauma show a markedly higher response rate to TEAS than those without a history of trauma.^23^ These findings suggest that a combination of EA and TEAS may be particularly suitable for victims of domestic violence and could effectively alleviate related psychiatric symptoms. This assessor‐blinded randomised controlled trial aimed to investigate whether adding EA + TEAS to care as usual (CAU) is superior to CAU alone in reducing depression and other psychiatric sequelae among these women.

## METHODS

### Design and settings

This trial was conducted between July 2022 and December 2024 in Teaching and Research Centre of School of Chinese Medicine, the University of Hong Kong, China. Participants were recruited through referrals from local domestic violence centres, including Harmony House, H.K.S.K.H. Lady Maclehose Centre, and Tung Wah Group of Hospitals Community Services Division, as well as through posters and social media. The study protocol was approved by the Institutional Review Board (IRB) of the University of Hong Kong/Hospital Authority Hong Kong West Cluster (UW 21‐238) and the Research Ethics Committee of Kowloon Central/Kowloon East (KC/KE‐22‐0072), and registered on www.clinicaltrials.gov (NCT05102253) prior to recruitment. All participants provided written informed consent before entering the trial. The study was reported in accordance with the CONSORT 2025 checklist.^26^

### Participants

Screening was conducted by an investigator (Sichang Yang) who had received clinical psychology and psychiatry training and was confirmed by a psychiatrist (Calvin Pak Wing Cheng).

The inclusion criteria were as follows: (i) Chinese women aged 18–65 years; (ii) had experienced domestic violence in the past 2 years, confirmed with the abuse assessment screen (AAS) questionnaire; (iii) were currently experiencing a major depressive episode according to the Diagnostic and Statistical Manual of Mental Disorders, Fifth Edition and (iv) had a Beck Depression Inventory‐II (BDI‐II) score of at least 14.^27^

The exclusion criteria included: (i) serious medical conditions or any life‐threatening situations that would limit participation; (ii) a history of brain injury or surgery; (iii) pregnancy or lactation; (iv) metal or electrical devices implanted in the body; (v) serious suicidal ideation or behaviours; (vi) a history of alcohol or drug abuse in the past year; (vii) a history of regular electrical AST or participation in an investigational intervention in the past 6 months or (viii) severe needle phobia.

### Randomisation and blinding

Participants were allocated to CAU alone (CAU group) or a combination of EA and TEAS (EA + TEAS group) in a ratio of 1:1 through block randomisation using randomly permuted block sizes of 4 or 6, generated by an independent research assistant. Allocation sequences were concealed using sequentially numbered opaque sealed envelopes, which were opened by treating clinicians only after baseline assessments were completed. To preserve blinding integrity, assessors (Wai Chun Mok, Kam Chi Yu and Pui Yan Chan) remained unaware of group assignments and participants were explicitly instructed not to disclose their treatment allocation to assessors during the study.

### Intervention

The intervention lasted 12 weeks and followed the treatment protocol.^28^ Although all participants were allowed to continue their existing routine care, participants in the EA + TEAS group additionally received two EA sessions at the clinic and three TEAS sessions at home per week, with no more than one session per day and no overlap between EA and TEAS on the same day. To minimise potential bias in the CAU group, participants in the CAU group were informed that EA + TEAS was part of the study protocol and that they would receive the same EA + TEAS regimen after completion of the 12‐week assessments. Post‐trial compensatory EA + TEAS treatment was provided accordingly. No other acupoint stimulation therapies were permitted during the study.

#### Care as usual

CAU mainly included psychotherapy, pharmacotherapy and advocacy interventions. The latter included shelter residence, informal counselling, legal assistance and social support.^29^ The most commonly prescribed psychotropic medications included antidepressants, anxiolytics/sedatives and antipsychotics. Psychotropic medication adjustments were at the discretion of the treating psychiatrists and general physicians during the study.

#### Electroacupuncture

EA was performed by registered Chinese Medicine practitioners (Sichang Yang, Xiaoru Hu and Mei Yan Chan) who had at least 3 years of clinical experience and had completed a training workshop for this study. The following six pairs of forehead acupoints were used in EA as previously reported^13^, ^19^, ^30^: Baihui (GV20, +) and Yintang (EX‐HN3, −), left Sishencong (EX‐HN1, −) and Toulinqi (GB15, +), right Sishencong (EX‐HN1, −) and Toulinqi (GB15, +), bilateral Shuaigu (GB8, L+, R−), bilateral Taiyang (EX‐HN5, L+, R−) and bilateral Touwei (ST8, L+, R−). The location and traditional Chinese medicine (TCM)‐based therapeutic rationale of these acupoints are described in Supporting Information S1: figure S1A and table S1.

Participants assumed a relaxed supine posture. Sterile, disposable acupuncture needles (0.22 mm in diameter and 25 mm in length) were inserted at a depth of 10–20 mm perpendicularly or obliquely into acupoints and manipulated to achieve the needling sensation. Electrical stimulation was then delivered through positive (+) and negative (−) electrode cord connections, as described above. A constant 2 Hz wave with 100 μs phase duration was delivered for 30 min using an ITO ES‐360 stimulator, with a peak output of 6 V and 48 mA. The stimulation intensity was adjusted to each participant's comfort level.

#### Transcutaneous electrical acupoint stimulation

Participants performed TEAS at home after completing a training workshop. The procedure targeted the bilateral Nei‐Guan (PC6) acupoints, following the protocol established in previous studies.^24^, ^25^ The participants lay or sat in a relaxed position. Briefly, constant‐current electrical impulses produced by a battery‐driven TEAS apparatus (SDP‐330, Yuwell, Suzhou Medical Appliances Co, Ltd.) were delivered to the bilateral acupoints through two adhesive electrode pads placed on the skin over the acupoints. The location and TCM‐based therapeutic rationale of PC6 are described in Supporting Information S1: figure S1B and table S1. Stimulation was administered at a frequency of 50 Hz with a pulse width that ramped from 30 to 100 μs and was then maintained at 100 μs throughout the 30‐min session. This frequency was chosen based on our preliminary experiments comparing the acceptability of different frequencies (2–100 Hz).^31^ Stimulation intensity was titrated to a ‘strong but comfortable’ level.

### Assurance of safety and treatment adherence

A hotline was established for participants to report adverse events (AEs) and treatment‐related concerns. Information about resources for victims of domestic violence was also provided, including university‐affiliated agencies, government‐ and nongovernmental organisation‐operated shelters and centres. To monitor adherence, participants were required to record their TEAS treatment on a designated log sheet. The date of each session, treatment‐related AEs and the number of missed and completed sessions were recorded. Participants were asked to bring the logs for investigators to verify the accuracy and completeness of their records. In addition, investigators regularly sent reminder messages to participants and asked them to show the updated log and/or to allow investigators to observe their TEAS treatment through WhatsApp.

### Assessments

To ensure consistency and accuracy of assessments throughout the study, designated assessors (Wai Chun Mok, Kam Chi Yu and Pui Yan Chan) attended pre‐ and mid‐trial training workshops. Senior psychiatrists who had extensive experience in clinical assessments provided training on the relevant rating instruments.

The primary outcome was baseline‐to‐endpoint (12 weeks) change in the severity of depression measured using the validated Chinese version of BDI‐II,^27^, ^32^ a 21‐item self‐report questionnaire with a score range of 0–63, where higher scores indicated greater severity of depressive symptoms. Scores of 0–13 corresponded to no symptoms, 14–19 corresponded to mild, 20–28 corresponded to moderate and ≥ 29 corresponded to severe depressive symptoms, respectively. This tool has been widely used to measure depressive symptoms among victimised women.^29^, ^33^

Multiple secondary outcomes were also measured, including changes in BDI‐II score from baseline to 3 and 6 weeks, clinical response and remission, defined as a ≥ 50% reduction in BDI‐II score from baseline and a BDI‐II score < 10, respectively. The 17‐item Hamilton Depression Rating Scale (HAMD‐17), an assessor‐rated questionnaire, was also used to measure the severity of depression.^34^ PTSD symptoms were measured using the PTSD Check List‐Civilian Version (PCL‐C).^35^, ^36^ Quality of sleep was measured using the Insomnia Severity Index (ISI).^37^, ^38^ The 10‐item Perceived Stress Scale (PSS‐10)^39^, ^40^, ^41^ and 12‐item Short Form Survey (SF‐12) (version 2)^42^, ^43^ were used to examine stress levels and health‐related quality of life, respectively. The SF‐12 encompasses both the mental component scale (MCS) and the physical component scale (PCS). Moreover, participants were asked to use the AAS to report any socio‐geographic changes and psychological, physical or sexual traumatic events that occurred.

All AEs were actively and systematically collected throughout the trial at each visit using standardised case report forms and were documented and categorised as either EA/TEAS‐related or nontreatment‐related. The severity of AEs was rated according to the Common Terminology Criteria for Adverse Events (v5.0) guidelines.^44^ Mild AEs were defined as insignificant and transient events that required no therapeutic intervention. Moderate AEs were those requiring minor, localised, or noninvasive therapeutic intervention. Severe AEs were those resulting in hospitalisation or prolonged hospitalisation and causing incapacity. Serious AEs triggered immediate reporting to the principal investigator and institutional review board to determine if additional actions were necessary.

Assessments using the BDI‐II, HAMD‐17, PCL‐C, ISI, PSS‐10, SF‐12 and AAS were conducted at baseline and at 3, 6 and 12 weeks. No interim outcome analyses were performed before the completion of the study to avoid assessment bias. In most cases, all assessments of a participant throughout the study were performed by the same assessor to ensure longitudinal consistency.

### Statistical analysis and data management

#### Estimation of sample size

The sample size was estimated based on our preliminary randomised controlled trials on TEAS and DCEAS.^25^, ^30^ Using previously established HAMD‐17 to BDI‐II conversion metrics,^45^ the expected between‐group difference in BDI‐II score was approximately 6.55. With a two‐sided 5% significance level (*α*), 80% power (1 − *β*), a conservatively assumed standard deviation of 10.8 and a dropout rate of 20%, 55 participants per group were required, giving a total target sample size of 110.

#### Data analysis

All data were double entered, stored and protected in accordance with the requirements of the Hong Kong Personal Data (Privacy) Ordinance (CAP 486). The analysis was performed on an intention‐to‐treat basis. Between‐group differences in continuous data on BDI‐II, HAMD‐17, PCL‐C, ISI, PSS‐10 and SF‐12 were tested using a linear mixed‐effects model with repeated measures and adjustment for baseline. The fixed effects included visit, treatment and the visit × treatment interaction, whereas individual participants were considered as random effects. Missing data for the primary outcome (BDI‐II) were imputed using multiple imputation under the missing‐at‐random assumption, followed by sensitivity analysis.

The clinical response and remission rates between the two groups at each visit were examined using the Chi‐square (*χ* ^2^) test, followed by logistic regression to obtain odds ratios with baseline adjustment. Categorical baseline variables and incidence of AEs were also analysed using the Chi‐square (*χ* ^2^) or Fisher's exact tests. The two‐sample *t*‐test and Wilcoxon rank–sum test were used to detect between‐group differences in continuous baseline variables. Subgroup analyses were conducted using mixed‐effects models to examine whether treatment effects on the primary outcome differed across major sociodemographic and clinical subgroups. Statistical significance was set at a two‐tailed level of *p* < 0.05. All statistical analyses were conducted using R software (version 4.4.1).

## RESULTS

### Baseline characteristics

**FIGURE 1:**
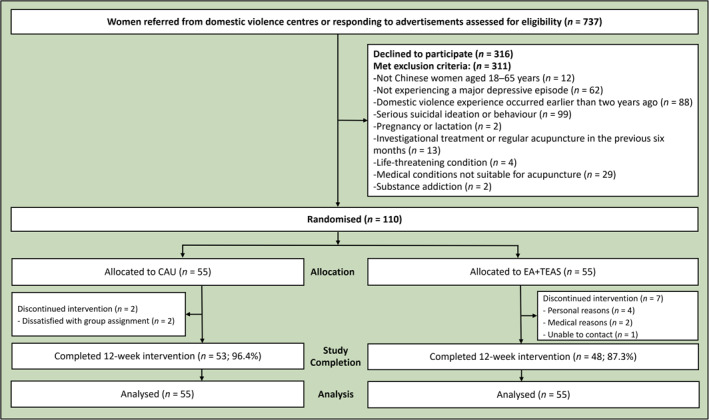
Study flowchart. All participants were analysed in their originally randomised groups based on the intention‐to‐treat principle. CAU, care as usual; EA, electroacupuncture; TEAS, transcutaneous electrical acupoint stimulation.

Of 737 participants assessed for eligibility, 110 were randomly assigned to the CAU or EA + TEAS group (*n* = 55 each) (figure 1). Overall, 91.8% (101/110) of participants completed the study, with a dropout rate of 3.6% (2/55) in the CAU group and 12.7% (7/55) in the EA + TEAS group. Among the 48 participants who completed the study in the EA + TEAS group, 91.7% (44/48) completed at least 75% of the assigned sessions of both EA and TEAS.

Baseline characteristics of the participants are presented in table 1. No statistically significant differences were observed in any baseline variables between the two groups. The median duration of domestic violence experienced was 6.0 years in the CAU group and 5.0 years in the EA + TEAS group. At study entry, 51.8% (57/110) of participants were living with their abusers, which remained stable throughout the trial (Supporting Information S1: table S2). Sixty percent (66/110) were receiving advocacy interventions, 28.2% (31/110) were receiving psychotherapy and 36.4% (40/110) were taking psychotropic medications at entry. Only minor adjustments to pharmacotherapy were made over the course of the study (Supporting Information S1: table S3). The severity of depressive symptoms was moderate to severe, with a median BDI‐II score of 28.0 in the CAU group and 34.0 in the EA + TEAS group.

**TABLE 1:** Baseline characteristics of study participants

Characteristic	CAU (*n* = 55)	EA + TEAS (*n* = 55)	Statistical values^a^	*p* value
Age, mean (SD), year	41.2 (10.7)	42.1 (12.7)	*t* = −0.398	0.691
BMI, median (IQR), kg/m^2^	21.7 (20.2–24.5)	21.8 (19.9–25.6)	*W* = 1464.500	0.774
Marital status, *n* (%)				
Single	18 (32.7)	20 (36.4)	*χ* ^2^ = 4.712	0.361
Living with spouse	0 (0.0)	2 (3.6)		
Not living with spouse	5 (9.1)	3 (5.5)		
Married	23 (41.8)	26 (47.3)		
Divorced	9 (16.4)	4 (7.3)		
Educational attainment, *n* (%)				
Primary school or below	0 (0.0)	2 (3.6)	*χ* ^2^ = 2.442	0.361
Secondary school	23 (41.8)	19 (34.5)		
Post‐secondary school or above	32 (58.2)	34 (61.8)		
Household monthly income (HK$), *n* (%)				
No income	6 (10.9)	6 (10.9)	*χ* ^2^ = 9.457	0.090
< 10 000	7 (12.7)	4 (7.3)		
10 000–20 000	6 (10.9)	18 (32.7)		
20 000–50 000	19 (34.5)	17 (30.9)		
50 000–100 000	13 (23.6)	9 (16.4)		
> 100 000	4 (7.3)	1 (1.8)		
Occupation, *n* (%)				
Professionals	11 (20.0)	12 (21.8)	*χ* ^2^ = 1.674	0.905
Technical staff	4 (7.3)	5 (9.1)		
Administration/management	13 (23.6)	9 (16.4)		
Housewife	15 (27.3)	17 (30.9)		
Student	5 (9.1)	7 (12.7)		
Others	7 (12.7)	5 (9.1)		
Living with abuser in the past month, *n* (%)	28 (50.9)	29 (52.7)	*χ* ^2^ = 0.036	0.849
Afraid of the abuser, *n* (%)	34 (61.8)	38 (69.1)	*χ* ^2^ = 0.643	0.423
Duration of domestic violence, median (IQR), year	6.0 (2.8–14.5)	5.0 (2.0–13.0)	*W* = 1576.500	0.701
Duration of depression, median (IQR), year	4.0 (1.5–8.0)	3.0 (1.5–6.0)	*W* = 1633.000	0.470
Family history of psychiatric disorders, *n* (%)	11 (20.0)	20 (36.4)	*χ* ^2^ = 3.638	0.056
Under advocacy intervention(s) at entry^b^, *n* (%)	37 (67.3)	29 (52.7)	*χ* ^2^ = 2.424	0.119
Under psychotherapy at entry, *n* (%)	19 (34.5)	12 (21.8)	*χ* ^2^ = 2.201	0.138
Under psychotropic medication at entry, *n* (%)	22 (40.0)	18 (32.7)	*χ* ^2^ = 0.629	0.428
Antidepressants	19 (34.5)	15 (27.3)	*χ* ^2^ = 0.681	0.409
Anxiolytics/sedatives	10 (18.2)	11 (20.0)	*χ* ^2^ = 0.059	0.808
Other psychotropic drugs	3 (5.5)	3 (5.5)	*χ* ^2^ = 1.000	1.000
Baseline BDI‐II score, median (IQR)^c^	28.0 (24.0–34.0)	34.0 (23.0–38.5)	*W* = 1224.000	0.084
Baseline HAMD‐17 score, mean (SD)^d^	21.1 (5.3)	21.3 (4.9)	*t* = −0.263	0.793
Baseline PCL‐C score, mean (SD)^e^	51.2 (9.4)	52.8 (10.3)	*t* = −0.882	0.380
Baseline PSS‐10 score, mean (SD)^f^	26.1 (4.0)	25.9 (4.4)	*t* = 0.226	0.822
Baseline ISI score, mean (SD)^g^	16.6 (5.1)	15.7 (4.6)	*t* = 1.001	0.319
Baseline SF‐12 score, mean (SD)^h^				
MCS	28.2 (8.1)	28.2 (8.3)	*t* = 0.048	0.962
PCS	43.0 (8.0)	42.7 (6.6)	*t* = 0.262	0.794

Abbreviations: BDI‐II, Beck Depression Inventory‐II; BMI, body mass index; CAU, care as usual; EA + TEAS, electroacupuncture + transcutaneous electrical acupoint stimulation; HAMD‐17, 17‐item Hamilton Depression Rating Scale; HK$, Hong Kong dollar; IQR, interquartile range; ISI, Insomnia Severity Index; MCS, mental component scale; PCL‐C, PTSD Check List‐Civilian Version; PCS, physical component scale; PSS‐10, 10‐item Perceived Stress Scale; PTSD, post‐traumatic stress disorder; SD, standard deviation; SF‐12, 12‐item Short Form Survey.

^a^ *t* for two sample *t*‐test; *χ* ^2^ for Pearson's Chi‐squared test; *W* for Wilcoxon rank sum test and *χ* ^2^ for tabular formatting for Fisher’s exact test for count data (where the final *p* value is derived from Fisher’s exact test, *χ*² is provided as this test does not inherently generate a test statistic).

^b^ Advocacy interventions included shelter residence, informal counselling, legal assistance and social support.

^c^ Range of possible scores is from 0 to 63. Higher scores indicate higher levels of depression.

^d^ Range of possible scores is from 0 to 52. Higher scores indicate higher levels of depression.

^e^ Range of possible scores is from 17 to 85. Higher scores indicate higher levels of PTSD.

^f^ Range of possible scores is from 0 to 40. Higher scores indicate higher levels of perceived stress.

^g^ Range of possible scores is from 0 to 28. Higher scores indicate higher levels of insomnia.

^h^ Range of possible scores is from 0 to 100, with higher scores indicating better health‐related quality of life.

### Depression

#### Primary outcome (BDI‐II)

**FIGURE 2:**
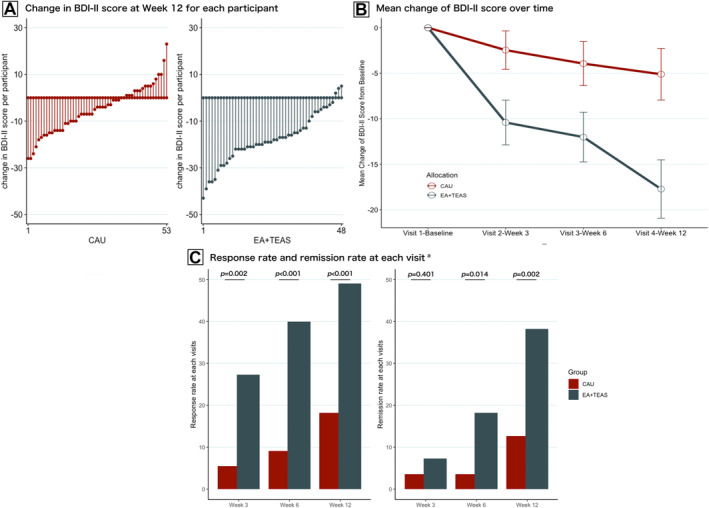
Changes in BDI‐II score, response rate and remission rate from baseline to 12 weeks. ^a^Response and remission are defined as a ≥ 50% reduction in BDI‐II score from baseline and a BDI‐II score < 10, respectively. Between‐group comparisons by Chi‐squared (*χ* ^2^) test. BDI‐II, Beck Depression Inventory‐II; CAU, care as usual; EA, electroacupuncture; TEAS, transcutaneous electrical acupoint stimulation.

After multiple imputation, the EA + TEAS group showed a 10.9‐point greater reduction in BDI‐II score than that of the CAU group at 12 weeks (95% confidence interval [CI] −16.5 to −5.4; *t* = −3.9, *p* < 0.001) (table 2, figure 2A, B). Sensitivity analysis consistently reinforced the superiority of the EA + TEAS group, including analyses restricted to participants with no modifications to ongoing psychotherapy or psychotropic medication use during the study (Supporting Information S1: tables S4 and S5). The greater reduction in the EA + TEAS group was also observed at 3 weeks (−8.0, 95% CI −11.3 to −4.8, *t* = −4.8, *p* < 0.001) and 6 weeks (−8.1, 95% CI −11.3 to −4.8, *t* = −4.8, *p* < 0.001).

**TABLE 2:** Changes in primary and secondary outcomes from baseline by groups

Outcomes	Change from baseline^a^		Between‐group difference	Statistics	*p* value
	CAU (*n* = 55)	EA + TEAS (*n* = 55)	EA + TEAS versus CAU		
Primary outcome					
BDI‐II^b^					
12 weeks	−5.1 (−7.4 to −2.8)***	−17.3 (−19.7 to −14.9)***	−10.9 (−16.5 to −5.4)^c^	*t* = −3.9	< 0.001
Secondary outcomes					
BDI‐II^b^					
3 weeks	−2.4 (−4.7 to −0.1)*	−10.4 (−12.7 to −8.0)***	−8.0 (−11.3 to −4.8)^d^	*t* = −4.8	< 0.001
6 weeks	−3.9 (−6.2 to −1.6)**	−12.0 (−14.3 to −9.6)***	−8.1 (−11.3 to −4.8)^d^	*t* = −4.8	< 0.001
HAMD‐17^b^					
3 weeks	−0.4 (−1.7 to 0.8)	−4.2 (−5.5 to −2.9)***	−3.8 (−5.6 to −2.0)^d^	*t* = −4.1	< 0.001
6 weeks	−1.1 (−2.3 to 0.2)	−5.1 (−6.4 to −3.8)***	−4.0 (−5.8 to −2.2)^d^	*t* = −4.4	< 0.001
12 weeks	−1.7 (−3.0 to −0.5)**	−9.0 (−10.4 to −7.7)***	−7.3 (−9.2 to −5.5)^d^	*t* = −7.9	< 0.001
PCL‐C^b^					
3 weeks	−0.9 (−3.6 to 1.7)	−8.5 (−10.9 to −6.0)***	−7.5 (−11.5 to −3.9)^d^	*t* = −4.1	< 0.001
6 weeks	−2.4 (−5.1 to 0.3)	−11.0 (−13.5 to −8.5)***	−8.6 (−12.2 to −5.0)^d^	*t* = −4.6	< 0.001
12 weeks	−5.7 (−8.4 to −3.1)***	−15.1 (−17.6 to −12.6)***	−9.3 (−13.0 to −5.7)^d^	*t* = −5.0	< 0.001
PSS‐10^b^					
3 weeks	−0.8 (−1.7 to 0.1)	−1.4 (−2.6 to −0.2)*	−0.6 (−2.1 to 0.9)^d^	*t* = −0.8	0.429
6 weeks	−1.6 (−2.5 to −0.6)**	−4.0 (−5.2 to −2.8)***	−2.4 (−3.9 to −0.9)^d^	*t* = −3.2	0.002
12 weeks	−1.9 (−2.8 to −1.0)***	−5.8 (−7.0 to −4.6)***	−3.9 (−5.4 to −2.4)^d^	*t* = −5.0	< 0.001
ISI^b^					
3 weeks	−0.1 (−1.5 to 1.2)	−3.6 (−4.8 to −2.4)***	−3.5 (−5.3 to −1.7)^d^	*t* = −3.8	< 0.001
6 weeks	−0.8 (−2.1 to 0.5)	−4.5 (−5.8 to −3.3)***	−3.8 (−5.6 to −1.9)^d^	*t* = −4.0	< 0.001
12 weeks	−1.1 (−2.4 to 0.2)	−6.2 (−7.4 to −4.9)***	−5.0 (−6.9 to −3.2)^d^	*t* = −5.4	< 0.001
SF‐12: MCS^e^					
3 weeks	2.6 (0.2–5.0)*	6.6 (4.4–8.8)***	4.0 (0.7–7.3)^d^	*t* = 2.4	0.018
6 weeks	4.8 (2.4–7.2)***	8.9 (6.7–11.1)***	4.1 (0.8–7.4)^d^	*t* = 2.4	0.016
12 weeks	5.7 (3.3–8.1)***	13.0 (10.7–15.3)***	7.4 (4.1–10.7)^d^	*t* = 4.3	< 0.001
SF‐12: PCS^e^					
3 weeks	0.0 (−1.9 to 1.9)	2.5 (0.8–4.2)**	2.5 (−0.0 to 5.0)^d^	*t* = 1.9	0.056
6 weeks	−0.9 (−2.8 to 1.0)	1.9 (0.2–3.6)*	2.8 (0.3–5.3)^d^	*t* = 2.1	0.033
12 weeks	−0.8 (−2.7 to 1.0)	2.6 (0.9–4.4)**	3.5 (0.9–6.0)^d^	*t* = 2.6	0.009

*Note*: Data are presented as mean (95% confidence interval).

Abbreviations: BDI‐II, Beck Depression Inventory‐II; CAU, care as usual; EA + TEAS, electroacupuncture + transcutaneous electrical acupoint stimulation; HAMD‐17, 17‐item Hamilton Depression Rating Scale; ISI, Insomnia Severity Index; MCS, mental component scale; PCL‐C, PTSD Check List‐Civilian Version; PCS, physical component scale; PSS‐10, 10‐item Perceived Stress Scale; PTSD, post‐traumatic stress disorder; SF‐12, 12‐item Short Form Survey.

^a^ *p < 0.05, **p < 0.01, ***p < 0.001, calculated using a mixed‐effects model with baseline adjustment to illustrate pre‐ and post‐treatment within‐group differences.

^b^ A greater negative value represents improvement in symptoms.

^c^ Estimated from mixed‐effects model with baseline adjustment and using multiple imputation to illustrate between‐group differences.

^d^ Estimated from mixed‐effects model with baseline adjustment to illustrate between‐group differences.

^e^ A greater positive value represents improvement in symptoms.

#### Hamilton Depression Rating Scale‐17

The EA + TEAS group exhibited a significant reduction in HAMD‐17 score from baseline at all three post‐baseline time points (3 weeks: −4.2, 95% CI −5.5 to −2.9; 6 weeks: −5.1, 95% CI −6.4 to −3.8; 12 weeks: −9.0, 95% CI −10.4 to −7.7; all *p* < 0.001), whereas the CAU group showed a significant reduction only at 12 weeks from baseline (−1.7, 95% CI −3.0 to −0.5, *t* = −2.7, *p* = 0.008). The EA + TEAS group showed a significantly greater reduction than the CAU group at all three post‐baseline time points (3 weeks: −3.8, 95% CI −5.6 to −2.0; 6 weeks: −4.0, 95% CI −5.8 to −2.2; 12 weeks: −7.3, 95% CI −9.2 to −5.5; all *p* < 0.001).

### PTSD and stress

#### PTSD Check List‐Civilian Version

The CAU group showed a significant reduction in PCL‐C score from baseline only at 12 weeks (−5.7, 95% CI −8.4 to −3.1, *t* = −4.3, *p* < 0.001). The EA + TEAS group showed a progressively greater reduction over time (3 weeks: −8.5, 95% CI −10.9 to −6.0; 6 weeks: −11.0, 95% CI −13.5 to −8.5; 12 weeks: −15.1, 95% CI −17.6 to −12.6; *p* values < 0.001). The EA + TEAS group showed markedly greater improvement than the CAU group at all three time points (3 weeks: −7.5, 95% CI −11.5 to −3.9; 6 weeks: −8.6, 95% CI −12.2 to −5.0; 12 weeks: −9.3, 95% CI −13.0 to −5.7; all *p* < 0.001).

#### Perceived Stress Scale‐10

Both groups showed a marked reduction in PSS‐10 score at 6 and 12 weeks from baseline. A significant reduction was additionally observed at 3 weeks in the EA + TEAS group (−1.4, 95% CI −2.6 to −0.2, *t* = −2.3, *p* = 0.021). The EA + TEAS group showed significantly greater improvement than the CAU group at 6 weeks (−2.4, 95% CI −3.9 to −0.9, *t* = −3.2, *p* = 0.002) and 12 weeks (−3.9, 95% CI −5.4 to −2.4, *t* = −5.0, *p* < 0.001).

### Quality of sleep and life

#### Insomnia Severity Index

A significant reduction in ISI score was observed at all three post‐baseline time points in the EA + TEAS group (3 weeks: −3.6, 95% CI −4.8 to −2.4; 6 weeks: −4.5, 95% CI −5.8 to −3.3; 12 weeks: −6.2, 95% CI −7.4 to −4.9; all *p* < 0.001) but not in the CAU group. The EA + TEAS group showed a significantly greater reduction in ISI score than the CAU group at all three time points (3 weeks: −3.5, 95% CI −5.3 to −1.7; 6 weeks: −3.8, 95% CI −5.6 to −1.9; 12 weeks: −5.0, 95% CI −6.9 to −3.2; all *p* < 0.001).

#### 12‐item Short Form Survey

The EA + TEAS group showed significant improvement on both MCS (3 weeks: 6.6, 95% CI 4.4–8.8; 6 weeks: 8.9, 95% CI 6.7–11.1; 12 weeks: 13.0, 95% CI 10.7–15.3; all *p* < 0.001) and PCS (3 weeks: 2.5, 95% CI 0.8–4.2; 6 weeks: 1.9, 95% CI 0.2–3.6; 12 weeks: 2.6, 95% CI 0.9–4.4; all *p* ≤ 0.030). The CAU group showed significant improvement on MCS only (3 weeks: 2.6, 95% CI 0.2–5.0; 6 weeks: 4.8, 95% CI 2.4–7.2; 12 weeks: 5.7, 95% CI 3.3–8.1; all *p* ≤ 0.037). The EA + TEAS group showed significantly greater improvement than the CAU group on MCS at all three time points (3 weeks: 4.0, 95% CI 0.7–7.3; 6 weeks: 4.1, 95% CI 0.8–7.4; 12 weeks: 7.4, 95% CI 4.1–10.7; all *p* ≤ 0.018) and PCS at 6 weeks (2.8, 95% CI 0.3–5.3, *t* = 2.1, *p* = 0.033) and 12 weeks (3.5, 95% CI 0.9–6.0, *t* = 2.6, *p* = 0.009).

### Response and remission

The response rates of the EA + TEAS group at all three time points were markedly greater than those of the CAU group (3 weeks: *χ* ^2^ = 9.6, 6 weeks: *χ* ^2^ = 14.2, 12 weeks: *χ* ^2^ = 11.8, all *p* ≤ 0.002), with 49.1% versus 18.2% (odds ratio: 5.0; 95% CI 2.0–12.2) at 12 weeks (Supporting Information S1: table S6, figure 2C).

The remission rate of the EA + TEAS group was significantly greater than that of the CAU group at 6 and 12 weeks (6 weeks: *χ* ^2^ = 6.0, 12 weeks: *χ* ^2^ = 9.4, all *p* ≤ 0.014), with 38.2% versus 12.7% (odds ratio: 6.9; 95% CI 2.4–20.3) at 12 weeks.

### Subgroup analyses

Subgroup analyses were performed to examine whether the effects of EA + TEAS on the primary outcome differed across major baseline variables (Supporting Information S1: table S7). A significantly greater reduction in BDI‐II score was observed among participants with a longer duration of depression, those currently living with abusers and those not receiving pharmacotherapy at entry.

### Adverse events

All treatment‐related AEs were mild and transient, and there were no significant differences between groups concerning major nontreatment‐related AEs (Supporting Information S1: table S8). For EA, bruising and headache (9.1% and 10.9%) were the most common AEs, whereas allergic symptoms (3.6%) were most frequent for TEAS.

## DISCUSSION

### Interpretation

This assessor‐blinded randomised controlled trial evaluated the effectiveness of a novel AST combining EA and TEAS for the treatment of depression and other psychiatric disorders in women who have experienced domestic violence.

Our findings demonstrate that the addition of EA + TEAS to CAU substantially improved depression, PTSD and insomnia. The between‐group differences at 12 weeks exceeded the minimal clinically important difference on all relevant scales^46^: 10.9 versus 5 points on the BDI‐II,^47^ 7.3 versus 3–5 points on the HAMD‐17,^48^ 5 versus 4 points on the ISI^49^ and 9.4 versus 7.9 points on the PCL‐C.^50^ EA + TEAS also markedly increased BDI‐II‐defined response and remission rates by approximately threefold at all three post‐baseline time points. Additionally, the EA + TEAS group showed significantly greater improvement than the CAU group on both perceived stress and quality of life, as measured by PSS‐10 and SF‐12. These effects appeared larger than those reported for acupuncture alone on PSS‐10 in patients with insomnia^51^ and on SF‐12 in patients with pain disorders.^52^, ^53^ Taken together, these results indicate that adjunctive EA + TEAS produces broad therapeutic benefits across domestic violence‐associated psychiatric disorders, particularly depression, PTSD and insomnia, and that combining TEAS with invasive acupuncture may yield additive and even synergistic effects.

Subgroup analyses revealed that participants living with abusers showed a greater response to EA + TEAS, suggesting that EA + TEAS may be more efficacious in those with ongoing exposure to domestic abuse. Depression can be categorised as ‘reactive’ (triggered by psychological trauma) or ‘endogenous’ (without clear triggers).^54^ Prior studies indicate that EA at PC6 reduces stress‐induced autonomic and neuroendocrine responses in rats,^55^ whereas transcutaneous nerve stimulation is more effective in reducing acute stress‐induced sympathetic responses in patients with PTSD and in improving PTSD with comorbid depression.^56^, ^57^ Together with our earlier findings that individuals with depression who had been exposed to psychological trauma responded more strongly to TEAS than those without,^23^ these results suggest that EA + TEAS may have particular therapeutic benefits for psychological trauma‐associated depression.

The EA protocol used in this study was based on DCEAS, a neuroanatomically grounded acupuncture approach previously developed by us.^13^, ^58^ DCEAS applies electrical stimulation to a dense cluster of acupoints located on the trigeminal nerve‐innervated areas of the scalp and forehead. The trigeminal sensory pathway has direct collateral connections with the brainstem reticular formation, particularly the raphe nuclei, where serotonin (5‐HT) is synthesised, and the locus coeruleus, where noradrenaline is produced.^13^ These two systems are extensively involved in the regulation of sleep, wakefulness, stress and mood.^59^, ^60^, ^61^ On the other hand, TEAS has been shown to enhance the synthesis and release of 5‐HT and noradrenaline in the brain,^62^ restore decreased levels of 5‐HT and catecholamines in patients^63^ and animal models of depression,^64^ and potentiate antidepressant effects.^65^ Both EA and TEAS have been shown to suppress hypothalamic–pituitary–adrenal axis hyperactivity.^13^ Their combined modulation of multiple neurochemical pathways, likely through additive or synergistic mechanisms, may partly explain the robust therapeutic effects observed with EA + TEAS.

In this study, participants in the EA + TEAS group showed high adherence to the intervention, with an attrition rate of only 12.7% (7/55). EA + TEAS‐related AEs were mild and transient. Given that approximately 50% of antidepressant users discontinue their medication mainly due to concerns about side effects^66^ and that abused women face various barriers to accessing medication and psychological therapy,^10^ EA + TEAS could serve as an effective, safe and accessible nonpharmacological option for this population.

### Limitations

This study has several limitations. First, CAU served as the control, rather than a sham EA + TEAS procedure, and participants were not blinded to group allocation. This may have introduced participant bias and influenced the reported outcomes. Second, the absence of an extended follow‐up beyond the 12‐week intervention period limited observation of longer‐term efficacy, warranting further investigation of sustained effects. Third, the generalisability of our results is limited by the culturally homogeneous sample of Chinese women. As coping patterns and responses to interventions can be culturally specific,^67^, ^68^ caution is warranted when generalising findings to other ethnic groups.

## CONCLUSION

The addition of EA + TEAS to CAU produced substantially greater improvements in depression and other psychiatric symptoms. EA + TEAS may serve as an effective intervention for women victims of domestic violence.

## AUTHOR CONTRIBUTIONS

Drafting of the manuscript: Sichang Yang. Revising the manuscript: Zhang‐Jin Zhang. Concept and design: Zhang‐Jin Zhang, Denise Shuk Ting Cheung, Agnes Tiwari, Calvin Pak Wing Cheng, Haiyong Chen and Xinjing Yang. Acquisition, analysis and interpretation of data: Zongshi Qin, Zhang‐Jin Zhang, Wai Chun Mok, Kam Chi Yu and Pui Yan Chan. Carrying out the treatments: Sichang Yang, Xiaoru Hu and Mei Yan Chan. Critical revision of the manuscript for important intellectual content: all authors. Obtained funding: Zhang‐Jin Zhang, Denise Shuk Ting Cheung, Agnes Tiwari, Calvin Pak Wing Cheng and Xinjing Yang. Administrative, technical, or material support: Wenqi Li, Sheng‐Nan Liu and Min‐Qing Gu. Referred participants: Doris Lee and Sai Ho Sin. Supervision: Zhang‐Jin Zhang.

## FUNDING

This study was funded by the Health and Medical Research Fund provided by the Government of the Hong Kong Special Administrative Region of China (Ref no: 18191451). The funder had no role in the design, data collection, data analysis, and reporting of this study.

## CONFLICT OF INTEREST STATEMENT

The authors declare no conflicts of interest.

## ETHICS STATEMENT

The authors assert that all procedures contributing to this work comply with the ethical standards of the relevant national and institutional committees on human experimentation and with the Helsinki Declaration of 1975, as revised in 2013. All procedures involving human subjects/patients were approved by the Institutional Review Board of the University of Hong Kong/Hospital Authority Hong Kong West Cluster (Ref no: UW 21–238) and the Research Ethics Committee of Kowloon Central/Kowloon East (Ref no: KC/KE‐22‐0072). Written informed consent was obtained from participants to participate in the study.

## Supporting information

Supporting Information S1

Supporting Information S2

## Data Availability

The data that support the findings of this study are available from the corresponding author, Zhang‐Jin Zhang, upon reasonable request. Data will be shared with researchers whose proposed use of the data has been approved and is in line with appropriate ethical, data sharing and open access principles. Analyses should be in line with relevant legislative, research funder and regulatory requirements, and after approval of a proposal.
