# Supplementary material for: Combining electroacupuncture and transcutaneous electrical acupoint stimulation for psychiatric disorders in women victims of domestic violence: An assessor‐blinded, randomised controlled trial
Source: Gen Psychiatr. 2026 Jul 7;39(4):e70035. doi: 10.1002/gps3.70035 (PMC13339927; doi:10.1002/gps3.70035)
Supplement: Supplementary file 1 — Supporting Information S1 [file GPS3-39-e70035-s001.pdf]

Supplementary materials

For

Combining electroacupuncture and transcutaneous electrical acupoint stimulation for psychiatric disorders in women victims of domestic violence: an assessor-blinded, randomised controlled trial

eTable 1. Location and traditional Chinese medicine (TCM)-based therapeutic effects of the acupoints used in the trial

eTable 2. Changes in proportion of participants living with abusers during the study

eTable 3. Adjustments of pharmacotherapy during the trial

eTable 4. Sensitivity analyses for the primary outcome

eTable 5. Sensitivity analyses for participants with no modifications to ongoing psychotherapy or psychotropic medication use during the study

eTable 6. Response and remission rates

eTable 7. Subgroup analyses of the EA+TEAS group in affecting baseline-to-endpoint BDI-II score

eTable 8. Incidence of adverse events, no. (%)

eFigure 1. The location of acupoints used.

This supplemental material has been provided by the authors to give readers additional information about their work.

eTable 1. Location and traditional Chinese medicine (TCM)-based therapeutic effects of acupoints used in EA and TEAS

Acupoint	Location	TCM-based therapeutic effects
**EA Acupoints**		
EX-HN3 (Yin-tang)	Located on the forehead, at the intersection point of the line connecting the two eyebrows and the midline of the forehead.	Calm the mind, soothe the spirit, and regulate mental health. Activate the trigeminal nerve, which in turn can modulate brain activity and neurotransmitter release. The trigeminal nerve has connections to various brain regions, including those implicated in emotional regulation and stress response. Stimulating Yin-tang is believed to help alleviate symptoms of stress, anxiety, insomnia, and promote relaxation.
GV20 (Bai-hui)	At the vertex of the head, located precisely 5 *cuns* directly above the midpoint of the frontal hairline.	Raises Yang, regulates the functions of the five Zangs, enhances cognitive function, alleviates stress, and demonstrates notable efficacy in addressing mental health conditions, specifically depression, insomnia, and anxiety.
EX-HN1 (Si-shen-cong), bilateral	At the vertex of the head, 1 *cun* laterally to each side of GV20 (Bai-hui).	Exhibit anxiolytic and calming effects, contribute to the inhibition of sympathetic nervous system functions, and are commonly employed in the treatment of depression, insomnia, and fatigue within clinical practice.
GB15 (Tou-lin-qi), bilateral	With eyes gazing straight ahead, the acupoint is situated directly above the pupils, 0.5 *cun* within the anterior hairline, at the midpoint of the line connecting the GV24 (Shen-ting) acupoint and the ST-8 (Tou-wei) acupoint.	Calming and mood-regulating effects. Stimulation of Tou-lin-qi aims to harmonize the flow of Qi and blood in the body, helping to alleviate stress and improve mental well-being.
GB8 (Shuai-gu, bilateral)	Situated directly superior to the apex of the ear, and positioned 1.5 *cun* within the hairline.	Reducing Qi stagnation and promoting meridian circulation. Clinically, it is commonly employed to alleviate symptoms related to stress, especially headaches and dizziness.
EX-HN5 (Tai-yang) bilateral	Located at the temple, within the depression approximately 1 *cun* posterior to the midpoint between the end of the eyebrow and the outer canthus of the eye.	Believed to regulate mental clarity and cognitive function. By promoting the circulation of Qi and blood to the head, Tai-yang nourishes the brain and contributes to mental well-being.
ST8 (Tou-wei) bilateral)	Located on the lateral aspect of the head, 0.5 *cun* above the hairline at the corner of the forehead, 4.5 *cuns* lateral to the midline of the head.	Have the effect of maintaining the normal function of the head and conveying Yang Qi to the head. Offering mood-regulating effects and are employed in the treatment of mental disorders within clinical practice.
**TEAS Acupoints**		
PC6 (Nei-guan, bilateral)	Located on the anterior forearm between the tendons of palmaris longus and flexor carpi radialis, at the junction of the distal sixth and proximal five sixths of the line connecting the middle points of the wrist and elbow crease.	Promote mental and emotional stabilization, alleviate pain, and broadly modulated cortical and limbic brain region activity. This point is often recommended for mental conditions like depression, anxiety and insomnia; and is widely used in clinical settings.

eTable 2. Changes in proportion of participants living with abusers during the study

	CAU	EA+TEAS	P value [a]
**Baseline**	**No. (%), n=55**	**No. (%), n=55**	
Did not live with abuser	27 (49)	26 (47)	0.849
Lived with abuser	28 (51)	29 (53)	
**3 Weeks**	**No. (%), n=52**	**No. (%), n=53**	
Did not live with abuser	29 (56)	23 (43)	0.205
Lived with abuser	23 (44)	30 (57)	
**6 Weeks**	**No. (%), n=52**	**No. (%), n=52**	
Did not live with abuser	29 (56)	26 (50)	0.556
Live with abuser	23 (44)	26 (50)	
**12 Weeks**	**No. (%), n=53**	**No. (%), n=48**	
Did not live with abuser	31 (58)	26 (54)	0.662
Live with abuser	22 (42)	22 (46)	

[a.] Calculated by Fisher's Exact Test for Count Data.

eTable 3. Adjustments of pharmacotherapy during the trial

CAU	EA+TEAS
**Increase in the variety of drugs**	
2 participants:<br>1. + Danxipress 10.5 mg (Flupentixol 0.5mg + Melitracen 10mg)<br>2. + Zolpidem 5 mg	1 participant:<br>1. + Desvenlafaxine 50 mg + Risperidone 1-2 mg
**Decrease in the variety of drugs**	
1 participant:<br>1. - Alprazolam PRN	No participant
**Decrease in the frequency/dosage**	
1 participant:<br>1. Venlafaxine 25 mg (baseline) to 12.5mg (12 weeks)	3 participants:<br>1. Lorazepam 1-2mg (baseline) to 0.5-2 mg PRN (12 weeks)<br>2. Zolpidem 5 mg (baseline) to Zolpidem 3-5 mg (12 weeks)<br>3. Duloxetine 30mg + Vortioxetine 10 mg + Diazepam 2.5 mg (baseline) to all PRN (12 weeks)
**Change in the variety/dosage of drugs**	
2 participants:<br>1. Escitalopram 20 mg (baseline) to Alprazolam 0.25mg (12 weeks)<br>2. Lexapro 10 mg + Brintellix 5 mg (baseline) to Vyvanse 20 mg (12 weeks)	1 participant:<br>1. Pregabaline 50 mg + Lisdexamfetamine 50 mg (baseline) to Pregabaline 75mg + Lisdexamfetamine 30 mg (12 weeks)

Each point represents a pharmacotherapy condition reported from one participant.

eTable 4. Sensitivity analyses for the primary outcome

Variable - mean (SD); n	CAU	EA+TEAS
Baseline BDI-II	29.3 (8.7); 55	32.3 (9.8); 55
Score Change (12 weeks)	-5.1 (10.3); 53	-17.7 (11.0); 48
Mean difference versus CAU (95% CI); *p* value [a]		-12.6 (-15.9, -9.3); <0.001
Mean difference versus CAU (95% CI); *p* value [b]		-12.2 (-15.5, -8.9); <0.001
Mean difference versus CAU (95% CI); *p* value [c]		-10.9 (-16.5, -5.4); <0.001
Score Change (baseline observation carried forward [d])	-4.7 (10.4); 55	-15.5 (12.8); 55
Mean difference versus CAU (95% CI); *p* value [d]		-10.9 (-14.3, -7.5); <0.001
Score Change (last observation carried forward [e])	-4.9 (10.1); 55	-15.5 (12.4); 55
Mean difference versus CAU (95% CI); *p* value [e]		-10.6 (-13.8, -7.3); <0.001

Abbreviations: CAU, care as usual; EA+TEAS, electroacupuncture + transcutaneous electrical acupoint stimulation; BDI-II, Beck Depression Inventory–II.

[a.] All observed cases.
[b.] Intention-to-treat dataset.
[c.] Multiple imputation.
[d.] Baseline observation carried forward applied to both groups.
[e.] Last observation carried forward was either baseline, 3 week or 6 week BDI-II score for the EA+TEAS group. For the CAU group last observation carried forward was the baseline BDI-II score.

eTable 5. Sensitivity analyses for participants with no modifications to ongoing psychotherapy or psychotropic medication use during the study

Variable<br>mean (SD), n	CAU	EA+TEAS	Difference in mean change from baseline to endpoint: EA+TEAS vs CAU (95% CI); P value
Baseline BDI-II	29.5 (8.8); 49	32.4 (9.8); 50	
12 Weeks BDI-II	24.3 (13.6); 47	14.4 (11.1); 44	
BDI-II changes	-5.3 (10.6); 47	-18.1 (10.9); 44	-12.5 (-16.0, -9.1); <0.001
Multiple Imputation			-11.5 (-16.6, -6.5); <0.001

eTable 6. Response and remission rates [a]

	CAU (n = 55)	EA+TEAS (n = 55)	Odds ratio (95% CI) [b]	Statistical value [c]	P value [c]
**Response rate (no.,%)**					
3 weeks	3 (5.5)	15 (27.3)	6.3 (1.7, 23.5)	9.6	0.002
6 weeks	5 (9.1)	22 (40.0)	7.7 (2.6, 23.0)	14.2	<0.001
12 weeks	10 (18.2)	27 (49.1)	5.0 (2.0, 12.2)	11.8	<0.001
**Remission rate (no., %)**					
3 weeks	2 (3.6)	4 (7.3)	2.8 (0.5, 17.0)	0.7	0.401
6 weeks	2 (3.6)	10 (18.2)	10.0 (1.9, 53.9)	6.0	0.014
12 weeks	7 (12.7)	21 (38.2)	6.9 (2.4, 20.3)	9.4	0.002

Abbreviations: CAU, care as usual; EA+TEAS, electroacupuncture + transcutaneous electrical acupoint stimulation.

a. The response and remission are defined as a ≥50% reduction in BDI-II score from baseline and a BDI-II score of less than 10, respectively. Subjects who discontinued treatment prior to having a post-baseline BDI-II score were considered non-responders and non-remitters.

b. Logistic regression with baseline adjustment was used for the calculation.

c. Chi-squared (χ^2) test was applied.

eTable 7. Subgroup analyses of the EA+TEAS group in affecting baseline-to-endpoint BDI-II score [a]

Subgroup	Mean difference between two subgroups (95% CI)	P value
**Age, years**		
≥ 45 vs. < 45	-4.2 (-10.8, 2.3)	0.217
**BMI**		
≥ 25 vs. <25	2.2 (-5.4, 9.8)	0.581
**Duration of depression, years**		
≥ 5 vs. < 5	-7.1 (-13.8, -0.4)	**0.041**
**Marital status**		
Married/living with partner vs. single	-3.4 (-13.0, 1.2)	0.497
Divorced/separated vs. single	-7.3 (-16.7, 2.2)	0.144
**Living with abusers in the past month**		
Yes vs. No	-7.9 (-14.3, -1.4)	**0.020**
**Under pharmacotherapy at entry**		
Yes vs. No	8.3 (1.6, 14.9)	**0.017**
**Under psychotherapy at entry**		
Yes vs. No	-5.6 (-13.0, 1.8)	0.149

Abbreviations: EA+TEAS, electroacupuncture + transcutaneous electrical acupoint stimulation; BMI, body mass index; BDI-II, Beck Depression Inventory–II.

[a.] Estimated from mixed-effects model with intention-to-treat dataset. Those whose statistical differences reached significance level (p<0.05) were highlighted with bold font.

eTable 8. Incidence of adverse events, no. (%)

Adverse event	CAU (n = 55)	EA+TEAS (n = 55)	P value [a]
Treatment-related adverse events			
Bruises in EA sites		5 (9.1)	
Nausea during EA		1 (1.8)	
Dizziness after EA		2 (3.6)	
Headache after EA		6 (10.9)	
Palpitation after EA		1 (1.8)	
Localized allergies after TEAS		2 (3.6)	
Localized numbness during TEAS		1 (1.8)	
Localized pain during TEAS		1 (1.8)	
Nausea after TEAS		1 (1.8)	
Headache after TEAS		1 (1.8)	
Palpitation after TEAS		1 (1.8)	
Major non-treatment-related adverse events [b]			
Cough/Cold	11 (20)	11 (20)	1.000
Chronic pain [c]	8 (14.5)	10 (18.2)	0.797
Headache	7 (12.7)	5 (9.1)	0.760
COVID-19	3 (5.5)	7 (12.7)	0.320
Traumatic injury/Sprain	7 (12.7)	2 (3.6)	0.161
Allergies	4 (7.3)	4 (7.3)	1.000
Sleep disturbance [d]	5 (9.1)	0 (0)	0.057

Abbreviations: CAU, care as usual; EA, electroacupuncture; TEAS, transcutaneous electrical acupoint stimulation.

a. Chi-squared (χ^2) test or Fisher's exact test were applied.

b. Only counted for those AEs recorded during assessments and by more than 5 patients in total.

c. Other than headache.

d. Including somnolence and insomnia.

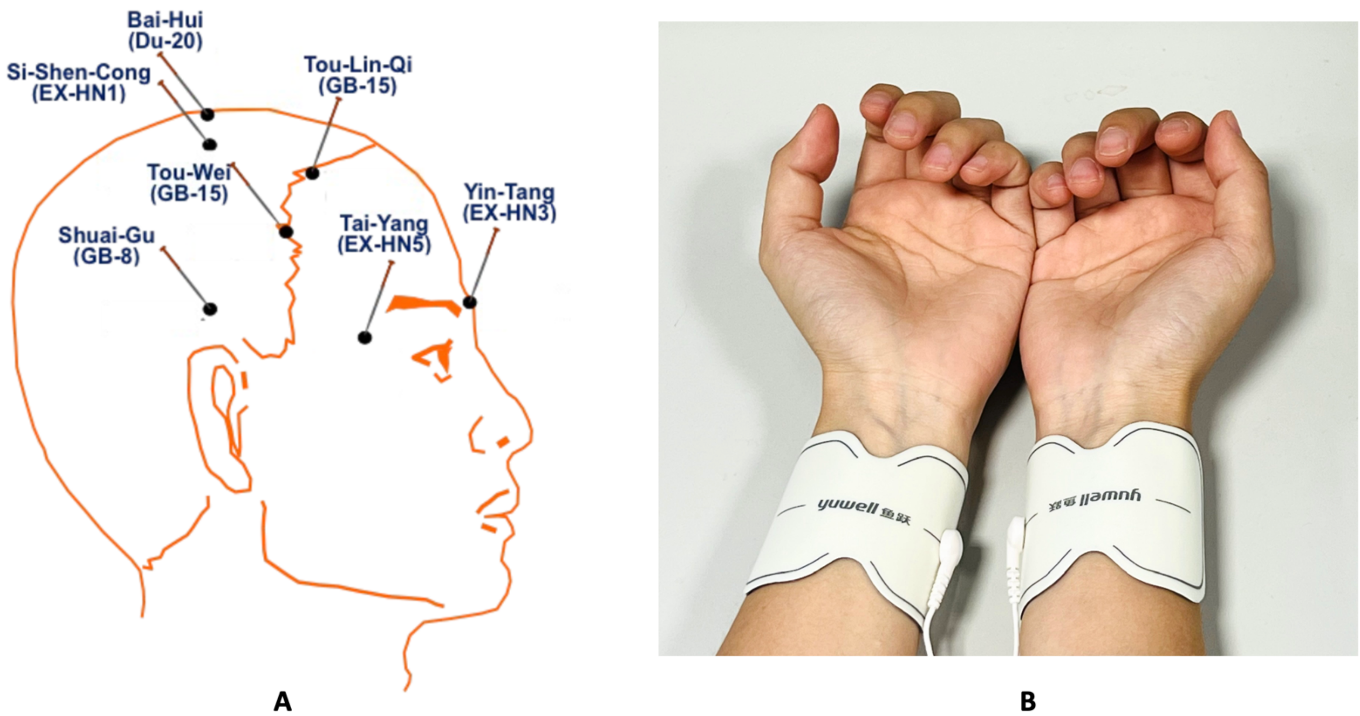

A B

eFigure 1. The location of acupoints used. (A) The location of acupoints used in electroacupuncture (EA) and (B) the placement of electrode pads on Nei-guan (PC6) for transcutaneous electrical acupoint stimulation (TEAS).
