# Supplementary material for: Combining electroacupuncture and transcutaneous electrical acupoint stimulation for psychiatric disorders in women victims of domestic violence: An assessor‐blinded, randomised controlled trial
Source: Gen Psychiatr. 2026 Jul 7;39(4):e70035. doi: 10.1002/gps3.70035 (PMC13339927; doi:10.1002/gps3.70035)
Supplement: Supplementary file 2 — Supporting Information S2 [file GPS3-39-e70035-s002.pdf]

Supplementary Material

Study protocol

Transcutaneous electrical acupoint stimulation and dense cranial electroacupuncture stimulation for psychiatric sequelae and related biomarkers in women victims of domestic violence: a protocol for a randomized controlled trial

Affiliated institution:

School of Chinese Medicine, LKS Faculty of Medicine, The University of Hong Kong

Principal Investigator for grant

Zhang-Jin Zhang, MD, PhD

School of Chinese Medicine, LKS Faculty of Medicine, The University of Hong Kong

E-mail: zhangzj@hku.hk

Confidentiality Statement

This document is the intellectual property of the Investigators. The information provided in this document is strictly confidential and is available for review to the sponsor, investigators, potential investigators, appropriate Ethics Committees, Investigational Review Boards, and other government regulatory bodies. No disclosure should take place without written authorization from the protocol developing investigators, except to the extent necessarily needed to obtain informed consent from potential subjects.

A. Introduction

Domestic violence and its psychiatric sequelae

Domestic violence, also referred to as intimate partner violence which overwhelmingly targets women of marriage and cohabitation, is a serious global public health issue with nearly 30% of women who have experienced physical or sexual abuse by their intimate partners.[1]

In Hong Kong, there were 4.5%-10% of women who experienced a physical violence and approximately 14,000 domestic violence cases reported to police offices each year.[2,3] Depression is the most common psychiatric sequela of domestic violence, with an average prevalence of 47.6% among women victims.[2] Our recent study has shown that 65.2% women survivors of intimate partner violence had mild to severe depression.[4] Abused women also often experienced post-traumatic stress disorder (PTSD), anxiety, substance abuse, and even suicidal thoughts. Although multiple psychological therapies, such as advocacy, cognitive behavioral therapy (CBT), and empowerment are a mainstay of domestic violence victim interventions,[5] a large portion of the victims could not achieve satisfactory response and have low acceptability for psychological interventions; this seems to be particularly apparent in Asian women.[6] Psychological interventions may be limited to patients with mild symptoms with long-term engagement of professionals. The development of non-psychological therapies is therefore highly desired.

Invasive and non-invasive acupuncture for depression and other stress trauma-related psychiatric symptoms

Acupuncture has been widely introduced into local clinics and hospitals of Hong Kong. Numerous studies have shown benefits and efficacy of acupuncture in the treatment of various psychiatric disorders, including depression and anxiety.[7,8] In addition to traditional invasive acupuncture in which needles are inserted into acupoints on the body, a non-invasive acupuncture mode called transcutaneous electrical acupoint stimulation (TEAS) also has been used in the treatment of postoperative anxiety, depression, autism, smoking cessation, and drug dependence.[9,10] Compared to invasive acupuncture, obviously, TEAS is more acceptable to patients and physicians as it is non-invasive, safer, and time-saving, without causing pain and needle phobia (Fig. 1).[9,10] Furthermore, TEAS can be performed by patients themselves at home. These suggest that a combination of invasive acupuncture and TEAS could serve as an effective and more acceptable intervention for the victims.

Our studies related to domestic violence and acupuncture

Over the past decade, we have evaluated the effectiveness of advocacy and Qigong interventions in domestic violence women victims.[2,4] We also have completed a series of epidemiological and methodological studies on women victims of domestic violence in local and international communities,[6,11,12] with over 30 related papers published. On the other hand, we have developed a novel acupuncture stimulation called dense cranial electroacupuncture stimulation (DCEAS) based on neuroanatomical rationales.[13] In this mode, electrical stimulation is delivered on dense acupoints located on the forehead innervated by the trigeminal nerve (Fig. 2A). Our several clinical trials have confirmed benefits and efficacy of DCEAS in patients with depression, obsessive-compulsive disorder (OCD), and neuropsychiatric sequelae of stroke.[14-17] Our two large-scale clinical trials have shown that patients with OCD and PTSD achieved an approximately 20% higher clinical remission rate on TEAS combined with CBT and/or antidepressants than those without TEAS.[9,10] These results provide a solid foundation for further evaluation of the effectiveness of combining TEAS and DCEAS.

Our studies on biomarkers associated with stress trauma and depression:

Our recent study has suggested that activity of leukocyte-containing telomerase, a ribonucleoprotein enzyme that maintains chromosome ends, increased following Qigong intervention in women victims of domestic violence.[4] The overexpression of platelet 5-HT1A receptors was correlated with more severe symptoms in patients with depressive disorder.[18] According to the presence and absence of stress trauma prior to its onset, depression can be classified into reactive and endogenous (melancholic) subtypes; domestic violence caused depression belongs to reactive subtype.[19] NALP3, standing for NACHT, LRR and PYD domains-containing protein 3, is an essential component of the inflammasome that modulates the release of cytokines, such as tumor necrosis factor (TNF), interleukin-4 (IL-4), IL-6, and IL-10. Our preliminary study has shown that leukocyte-containing NALP3 was significantly lower in patients with reactive depression than healthy controls and endogenous subtype and the serum brain-derived neurotrophic factor (BDNF) level was significant negatively correlated with the severity of reactive depression.

Determining relationships between potential biomarkers and the severity of psychiatric symptoms will lead us to better understand the pathogenesis of stress trauma and biochemical mechanisms of acupuncture effects.

B. Hypothesis and Aims to be Tested

We hypothesize that additional therapy with a combination of TEAS and DCEAS produces better outcomes than routine care alone in improving depression and other psychiatric sequelae of domestic violence; the therapeutic effects of acupuncture may be associated with varying modulation of blood biomarkers. To test this hypothesis, an assessor-blinded, randomized controlled trial will be conducted to pursue the following the two aims: (1) to compare the effectiveness of routine care with and without additional TEAS and DCEAS in treating depression, and other psychiatric symptoms of domestic violence women victims; and (2) to determine baseline-to-endpoint changes in levels of blood biomarkers, and their correlations with clinical outcomes.

C. Plan of Investigation

Outline: This is an assessor-blinded, randomized controlled trial. A total of 110 women victims of domestic violence aged 18-65 years will be recruited from local community centers and domestic violence shelters (refugee centers). They will be randomly assigned to routine care combined with (n = 55) and without (n = 55) additional acupuncture (TEAS+DCEAS) for 12 weeks. The study flow chart are presented in Fig. 3. Acupuncture therapy will be conducted with 2 DCEAS sessions at clinics and 3 TEAS sessions at home each week. The primary outcome is the Beck Depression Inventory II (BDI-II) for depression. Secondary outcomes include the 17-item Hamilton Depression Rating Scale (HAMD-17) for depression, Perceived Stress Scale (PSS) for stress, PTSD Check List-Civilian Version (PCL-C) for PTSD symptoms, Insomnia Severity Index (ISI) for sleep and 12-Item Short Form Survey (SF-12) for quality of life. Two 10-ml blood samples will be drawn respectively at baseline and at the end of 12-week study for measurement of blood biomarkers, respectively. The SPIRIT diagram of all assessments at enrolment, allocation, and different time points can be found in Fig. 4.

(i) Subjects

(a) Inclusion criteria: Subjects will be eligible for this study if they: (1) are Chinese women aged 18-65 years; (2) have experienced domestic violence in the previous two years,

confirmed with the Abuse Assessment Screen (AAS) Screen Questionnaire; and (3) are currently experiencing a major depressive episode according to the criteria defined in the Diagnostic and Statistical Manual of Mental Disorders, fifth edition (DSM-5), with a BDI-II score of at least 14.

(b) Exclusion criteria: Subjects will be excluded if they have: (1) serious medical conditions or any life-threatening situation that may limit their participation; (2) a history of brain injury or surgery; (3) pregnancy or lactation; (4) investigational intervention in the previous 6 months; (5) heart pacemaker or other metal or electrical devices implanted in the body; (6) serious suicidal ideation or behaviours; (7) history of alcohol or drug abuse in the past year; (8) history of regular electrical acupoint stimulation in previous 6 months; or (9) severe needle phobia.

(c) Estimation of sample size:

The sample size for this trial was estimated based on our preliminary trials [9, 15]. According to previous established HAMD-17 to BDI-II conversion metrics [20],the results indicating an approximately difference of 6.55 in the BDI-II score between two groups. A sample size of 55 in each group will be sufficient with a 95% level of significance (α), 80% power (1 − β), and a conservatively assumed standard deviation of 10.8, and a dropout rate of 20%. Therefore, this trial will include 110 subjects.

(ii) Methods

(a) Screening, randomization, and blindness: Screening will be conducted by social workers, nurses, general physicians, registered occupational therapists, and research assistants; and further confirmed by a psychiatrist. One senior research assistant will be employed in charge for randomization and central allocation. Random codes will be produced in advance using simple, complete, non-sequential random codes with block sizes of 4 or 6. Central allocation will be conducted, i.e., site investigators who are responsible for confirming subjects' eligibility will call the coordinator to obtain a random code for an eligible subject. Allocation sequences were concealed using sequentially numbered, opaque, sealed envelopes, which were opened by treating clinicians only after the completion of baseline assessments. Participants will be allocated to routine care with or without TEAS+DCEAS with a ratio of 1:1. Treatment conditions will be blind to clinical assessors.

(b) Routine care: All participants of the two groups will continue their current routine care as usual. The routine care may include medications, advocacy intervention, and CBT.

(c) DCEAS regimen: DCEAS will be conducted for 2 sessions per week for 12 consecutive weeks. A brief introduction of acupuncture procedure will be given by a registered Chinese medicine practitioner (RCMP) in the first visit. If possible, all sessions of acupuncture treatment for a participant will be performed by the same registered Chinese medicine practitioner if possible in order to eliminate deviations from different acupuncturists.

Electrical stimulation will be conducted on six pairs of forehead acupoints with positive (+) and negative (-) electrode cord connection as follows (Fig. 2B): Bai-Hui (GV20, +) and Yin-Tang (EX-HN3, -), left Si-Shen-Cong (EX-HN1, -) and Tou-Lin-Qi (GB15, +), right Si-Shen-Cong (EX-HN1, -) and Tou-Lin-Qi (GB15, +), bilateral Shuai-Gu (GB8, L+, R-), bilateral Tai-Yang (EX-HN5, L+, R-), and bilateral Tou-Wei (ST8, L+, R-).

Disposable acupuncture needles (0.22 mm in diameter and 25 mm in length) will be inserted at a depth of 10-20 mm perpendicularly or obliquely into acupoints. Manual manipulation will be carried out for all acupoints to evoke needling sensation, followed by electrical stimulation. The output peak current and voltage of the machine (model: ITO ES-360) are 6 V and 48 mA, respectively, with constant wave at frequency of 2 Hz and phase duration of 100 μs for 30 min. The stimulation intensity will be adjusted to a level at which patients felt most comfortable. The low frequency could produce broader neuromodulation compared to higher frequency. Each session of treatment will last 30 minutes.

(d) TEAS regimen: TEAS will be done for 3 times per week for 12 consecutive weeks by participants at home. A training workshop will be conducted to instruct participants how to use TEAS stimulator. Briefly, TEAS will be delivered on the bilateral Nei-Guan (PC6), which is located on the anterior forearm, between the tendons of the palmaris longus and flexor carpi radialis, at the junction of the distal sixth and proximal five sixths of the line connecting the middle points of the wrist and elbow crease (Fig. 1). This acupoint is specifically beneficial for insomnia, sadness, nervousness, palpitation, chest congestion, nausea and vomiting according to traditional Chinese medicine theory. Neuroimaging has shown that acupuncture stimulation on Nei-Guan can modulate a wide range of cortical and limbic brain region activity.

Constant current electrical impulses will be produced from a TEAS apparatus (SDP-330, Yuwell, Suzhou Medical Appliances Co, Ltd., Suzhou, China). Two electrode pads will be adhered onto the acupoint skin. The stimulation frequency will be set at 50 Hz with a pulse width increasing from 30-100 μs, and keeping in 100 μs at treatment stage. This frequency is the most comfortable and also has been shown to robustly induce the release of endogenous opiate neuropeptides in the central nervous system. The pulse amplitude will be adjusted to a level at which the perception of 'strong but comfortable' is achieved. Each session of treatment will last 30 minutes.

(e) Post-trial treatment of subjects of routine care group: Those who are initially assigned to routine care group will have a choice to receive TEAS and/or DCEAS treatment for 12 weeks after they complete the trial. The post-trial treatment will serve as a compensation for their participation.

(f) Termination criteria and post-termination management: To ensure participants' safety, those who cannot tolerate TEAS or DCEAS or are hospitalized due to the aggravation of their condition will be discontinued from the study and given individual clinical treatment.

(iii) Study design

(a) Clinical assessment: The primary outcome will be evaluated using BDI-II, one of the most widely used psychometric tests for measuring the severity of stress trauma-related depression.[21] The secondary outcomes are clinician-assessed depression, stress, PTSD symptoms, insomnia and quality of life that will be assessed with HAMD-17, PSS, PCL-C, ISI and SF-12, respectively. These instruments have been widely used in domestic violence studies. Adverse events will be assessed using the standardized case report forms. The aforesaid instruments have been extensively implemented in our previous studies. Assessment will be performed at baseline, Week 3, Week 6, and Week 12. The reason for Week 3 as the earliest measurement point is based on our previous TEAS and DCEAS studies, showing that the significant improvement was observed at as early as at Week 3.

(b) Maintenance of fidelity and consistency: As participants will conduct TEAS themselves at home, they are required to record their TEAS sessions in a log sheet on the daily basis. Meanwhile, we will send a reminder to participants by mobile phone, monitor their TEAS treatment, answer their questions, and receive their emergency report.

The study will last about 3 years and be carried out in multiple sites. There also will be many different professional investigators involved in the study. To ensure consistency of DCEAS and assessment across sites and throughout a study period, training workshop will be conducted at pre-trial and once per year thereafter. All investigators and caregivers will receive training. Training manual will be prepared. For DCEAS, the PI and a senior acupuncturist will instruct and demonstrate DCEAS. Trainees are required to obtain satisfactory training outcomes which will be rated by the PI and a senior acupuncturist. For clinical assessment training, an investigator who has extensive experience in clinical assessment will instruct clinical instruments. A depressed patient video will be used for the measurement of BDI-II. An inter-rater reliability coefficient (k value) of >0.80 must be achieved after the completion of each training workshop.

To further ensure consistency and fidelity, the following additional measures will be taken: (1) All assessments from baseline throughout endpoint for each participant will be performed, if possible, by the same assessor who is blind to participants' intervention; (2) all DCEAS sessions of each patient will be conducted by the same acupuncturist, if possible; and (3) biomarker analysis will be done by the same research assistant.

(c) Safety assessment, assurance and ethical consideration:

For each visit to treatment, an standardized case report form will be used to record patients' AEs, including whether any AE has occurred, occurrence time, duration, type of event, severity, management, and its outcome. Also, whether the adverse events are caused by acupuncture would be assessed. The Common Terminology Criteria for Adverse Events (CTCAE) v5.0 criteria will be used to evaluate the severity of each adverse event [22]. All associated research staffs are trained to recognize and respond to all the adverse events. If an adverse event is regarded as a serious adverse event, it will be reported to project investigators (ZJZ) and Ethics Committee to review immediately. They will decide whether the patient will be temporarily/permanently interrupted from their current treatment, whether

the patient will withdraw from this clinical trial and whether this trial needs to be adjusted or terminated.

Case Record Form (CRF) will be used to record patients' information, assessment, and treatment. In addition to mobile phone, emergency contact numbers will be provided to participants for them to report emergency and seek advice. Ethical approval will be obtained before the recruitment starts. All participants are required to provide written consent. The proposed study will be registered in www.clinicalTrials.gov.

(iv) Data processing and analysis

(a) Data management

Data will be collected by blinded independent assessors and entered into a password-protected computer using a double entry method. Each participant will be assigned an identification number for data processing and communication. Personal data will be stored and protected in accordance with the requirements of the Hong Kong Personal Data (Privacy) Ordinance (CAP 486). The deidentified data will be used for analysis and publication. The publication of any results will not reveal the identity of any participant. The results will be provided to the participants and social workers in a short summary after the publication of the trial if required. To prevent patient attrition, research assistants will confirm appointment information with participants and send a reminder before the scheduled appointment. In addition, a transport allowance will be provided to each participant upon completion.

(a) Data analysis

One biostatistician who is blinded to interventions will be responsible for statistical analysis. Descriptive statistics tables will be used to present basic information on all variables. For continuous variables, the mean and standard deviation (or medians, 25th, and 75th percentiles) will be presented; while for categorical variables, the number of participants in each category and its percentage will be presented separately. The analysis will be carried out on the intention-to-treat (ITT) population. All eligible participants will be included in the analysis. Missing data on the primary outcome will be imputed using the multiple imputation method under the missing at random (MAR) assumption follow by sensitivity analysis.

The primary outcome will be analyzed by a linear mixed-effects model with repeated-measures, with adjustment for the baseline; visit, treatment, and the visit × treatment interaction as the fixed effect, and individual subject as the random effect. A significant two-way interaction will be considered to demonstrate whether the treatment group has a greater change in the BDI-II score from baseline to assessing points than the routine care group. The effect of the treatment will be estimated by the difference between treatments, and will be presented together with its associated 95% confidence intervals (CIs). A similar approach will be applied to other continuous outcomes.

For the response rate and remission rate, logistic regression, with adjustment for baseline scores, and Chi-square (χ^2) test will be used. The definition of responder is subjects with ≥50% reduction from the baseline BDI-II score; the definition of remission is subjects with BDI-II score less than 10. Subjects who discontinue treatment prior to having a post-baseline BDI-II score will be considered non-responders/not remission.

Subgroup analysis will be further conducted to detect whether demographic and baseline clinical variables are associated with outcomes. Student t-test and Wilcoxon rank sum test will be used to detect differences in continuous baseline variables and biomarker levels between the two groups. Categorical baseline variables, including discontinuation and incidence of adverse events will be analyzed using Chi-square (χ^2) test or Fisher's exact tests. Linear mixed-effects model will be conducted to examine intercorrelations between clinical outcomes and biomarker levels and among biomarker levels. Statistical significance is defined as a two-tailed $P < 0.05$.

D. Anticipated results and potential pitfalls

Our previous studies have confirmed the effectiveness of TEAS and DCEAS in patients with depression, PTSD, and OCD.[9,10,14-17] We also have done a series of intervention, epidemiological, and methodological studies in domestic violence women victims. These provide a direct and solid basis to ensure the success of the proposed study. Additional acupuncture is expected to produce the superior efficacy than routine care alone in improving psychiatric symptoms occurred in stress trauma of domestic violence. Multiple correlations between outcomes and biomarkers would be identified.

Although local collaborative network, protocols, and research team have been well established, according to our previous studies, slow recruitment progress often happened due to unpredictable causes. If the recruitment could not reach the goal as scheduled timeline at the end of the first year, additional study sites and promotion plan will be sought.

E. Impact on People's Health and Health Services as well as Plan to Disseminate Research Findings to End Users

Women victims of domestic violence are a socially vulnerable population who need more intervention options in addition to routine cares. If the proposed study could demonstrate that acupuncture with a combination of TEAS and DCEAS is effective in treating violence trauma-caused psychiatric sequelae, we will introduce TEAS and DCEAS to physicians, Chinese medicine practitioners, nurses, social workers, and other caregivers of local communities and domestic violence centers via holding training workshops. Through these training workshop, TEAS+DCEAS could serve as an additional intervention for domestic violence victims.

Key References

1. World Health Organization. Global and regional estimates of violence against women: prevalence and health effects of intimate partner violence and non-partner sexual violence. Geneva: World Health Organization, 2013.
2. Tiwari A, Fong DY, Yuen KH, Yuk H, Pang P, Humphreys J, Bullock L. Effect of an advocacy intervention on mental health in Chinese women survivors of intimate partner violence: a randomized controlled trial. JAMA. 2010;304(5):536-43.
3. https://www.edb.gov.hk/attachment/tc/teacher/student-guidance-discipline-services/lecture-notes/lecture-notes-201718/20180103_Identification_and_intervention.pdf
4. Cheung DST, Deng W, Tsao SW, Ho RTH, Chan CLW, Fong DYT, Chau PH, Hong AWL, Fung HYKY, Ma JLC, Tiwari AFY. Effect of a Qigong Intervention on Telomerase Activity and Mental Health in Chinese Women Survivors of Intimate Partner Violence: A Randomized Clinical Trial. JAMA Netw Open. 2019;2(1):e186967.
5. Hackett S, McWhirter PT, Lesher S. The Therapeutic Efficacy of Domestic Violence Victim Interventions. Trauma Violence Abuse. 2016;17(2):123-32.
6. Sun KS, Lam TP, Piterman L, Lam KF, Tang WS, Kwok KW, Chan HY, Wu D, Tiwari A. Management of Domestic Violence by Primary Care Physicians in Hong Kong: Association With Barriers, Attitudes, Training, and Practice Background. J Interpers Violence. 2019;17:886260519869067.
7. Amorim D, Amado J2, Brito I2, Fiuza SM3, Amorim N3, Costeira C4, Machado J5. Acupuncture and electroacupuncture for anxiety disorders: A systematic review of the clinical research. Complement Ther Clin Pract. 2018;31:31-37.
8. Smith CA, Armour M, Lee MS, Wang LQ, Hay PJ. Acupuncture for depression. Cochrane Database Syst Rev. 2018;3:CD004046.
9. Feng B, Zhang Y, Luo LY, Wu JY, Yang SJ, Zhang N, Tan QR, Wang HN, Ge N, Ning F, Zheng ZL, Zhu RM, Qian MC, Chen ZY, Zhang ZJ. Transcutaneous electrical acupoint stimulation for post-traumatic stress disorder: Assessor-blinded, randomized controlled study. Psychiatry Clin Neurosci. 2019;73(4):179-186
10. Feng B, Zhang ZJ, Zhu RM, Yuan GZ, Luo LY, McAlonan GM, Xu FZ, Chen J, Liu LY, Lv YY, Wong HK, Zhang Y, Zhu LX. Transcutaneous electrical acupoint stimulation as an adjunct therapy for obsessive-compulsive disorder: A randomized controlled study. J Psychiatr Res. 2016;80:30-37.
11. Greene MC, Rees S, Likindikoki S, Bonz AG, Joscelyne A, Kaysen D, Nixon RDV, Njau T, Tankink MTA, Tiwari A, Ventevogel P, Mbwambo JKK, Tol WA. Developing an integrated intervention to address intimate partner violence and psychological distress in Congolese refugee women in Tanzania. Confl Health. 2019;13:38.
12. Wong JY, Tiwari A, Fong DY, Bullock L. A Cross-Cultural Understanding of Depression Among Abused Women. Violence Against Women. 2016;22(11):1371-96.
13. Zhang ZJ, Wang XM, McAlonan GM. Neural acupuncture unit: a new concept for interpreting effects and mechanisms of acupuncture. Evid Based Complement Alternat Med. 2012;2012:429412.
14. Zhang ZJ, Ng R, Man SC, Li TY, Wong W, Tan QR, Wong HK, Chung KF, Wong MT, Tsang WK, Yip KC, Ziea E, Wong VT. Dense cranial electroacupuncture stimulation for major depressive disorder--a single-blind, randomized, controlled study. PLoS One. 2012;7(1):e29651.
15. Zhang ZJ, Zhao H, Jin GX, Man SC, Wang YS, Wang Y, Wang HR, Li MH, Yam LL, Qin ZS, Yu KT, Wu J, Ng FB, Ziea TE, Rong PJ. Assessor- and participant-blinded, randomized controlled trial of dense cranial electroacupuncture stimulation plus body

acupuncture for neuropsychiatric sequelae of stroke. Psychiatry Clin Neurosci. 2019 Nov 20. doi: 10.1111/pcn.12959. [Epub ahead of print].

16. Zhang ZJ, Wang XY, Tan QR, Jin GX, Yao SM. Electroacupuncture for refractory obsessive-compulsive disorder: a pilot waitlist-controlled trial. J Nerv Ment Dis. 2009;197(8):619-22.
17. Man SC, Hung BH, Ng RM, Yu XC, Cheung H, Fung MP, Li LS, Leung KP, Leung KP, Tsang KW, Ziea E, Wong VT, Zhang ZJ. A pilot controlled trial of a combination of dense cranial electroacupuncture stimulation and body acupuncture for post-stroke depression. BMC Complement Altern Med. 2014;14:255.
18. Zhang ZJ, Wang D, Man SC, Ng R, McAlonan GM, Wong HK, Wong W, Lee J, Tan QR. Platelet 5-HT(1A) receptor correlates with major depressive disorder in drug-free patients. Prog Neuropsychopharmacol Biol Psychiatry. 2014;53:74-9.
19. Malki K, Keers R, Tosto MG, Lourdusamy A, Carboni L, Domenici E, Uher R, McGuffin P, Schalkwyk LC. The endogenous and reactive depression subtypes revisited: integrative animal and human studies implicate multiple distinct molecular mechanisms underlying major depressive disorder. BMC Med. 2014;12:73.
20. Furukawa TA, Reijnders M, Kishimoto S, Sakata M, DeRubeis RJ, Dimidjian S, et al. Translating the BDI and BDI-II into the HAMD and vice versa with equiper- centile linking. Epidemiol Psychiatr Sci. 2019;29:e24.
21. Barbano AC, van der Mei WF, deRoon-Cassini TA, Grauer E, Lowe SR, Matsuoka YJ, O'Donnell M, Olff M, Qi W, Ratanatharathorn A, Schnyder U, Seedat S, Kessler RC, Koenen KC, Shalev AY; International Consortium to Prevent PTSD. Differentiating PTSD from anxiety and depression: Lessons from the ICD-11 PTSD diagnostic criteria. Depress Anxiety. 2019;36(6):490-498.
22. Health NIo. Common terminology criteria for adverse events (CTCAE) v5. 0. NIH, National Cancer Institute 2017.

Fig. 1. Transcutaneous Electrical Acupoint Stimulation (TEAS) on bilateral Nei-Guan (PC6) acupoints.

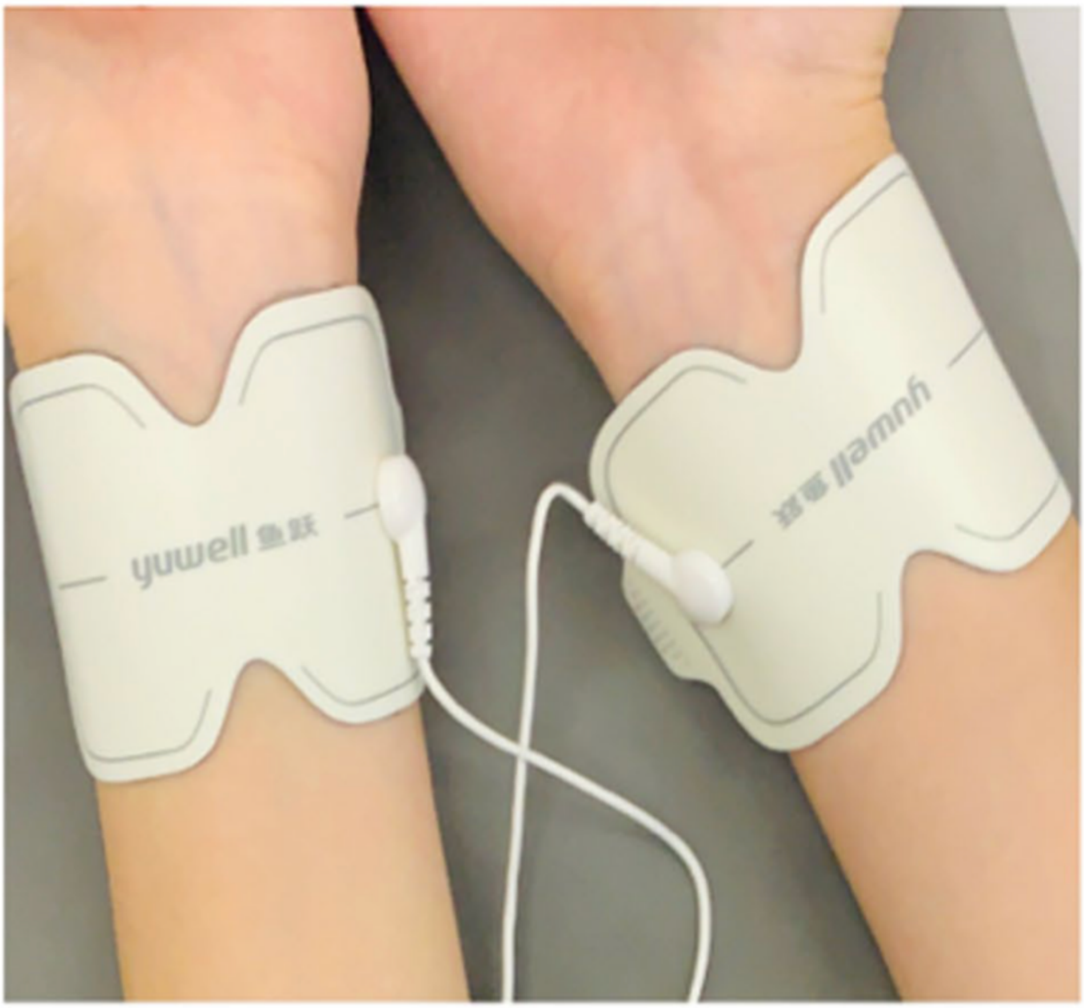

Fig. 2. Putative neuropathways for dense cranial electroacupuncture stimulation (DCEAS) (A) and the forehead acupoints used in the proposed study is less than that shown in plot A.

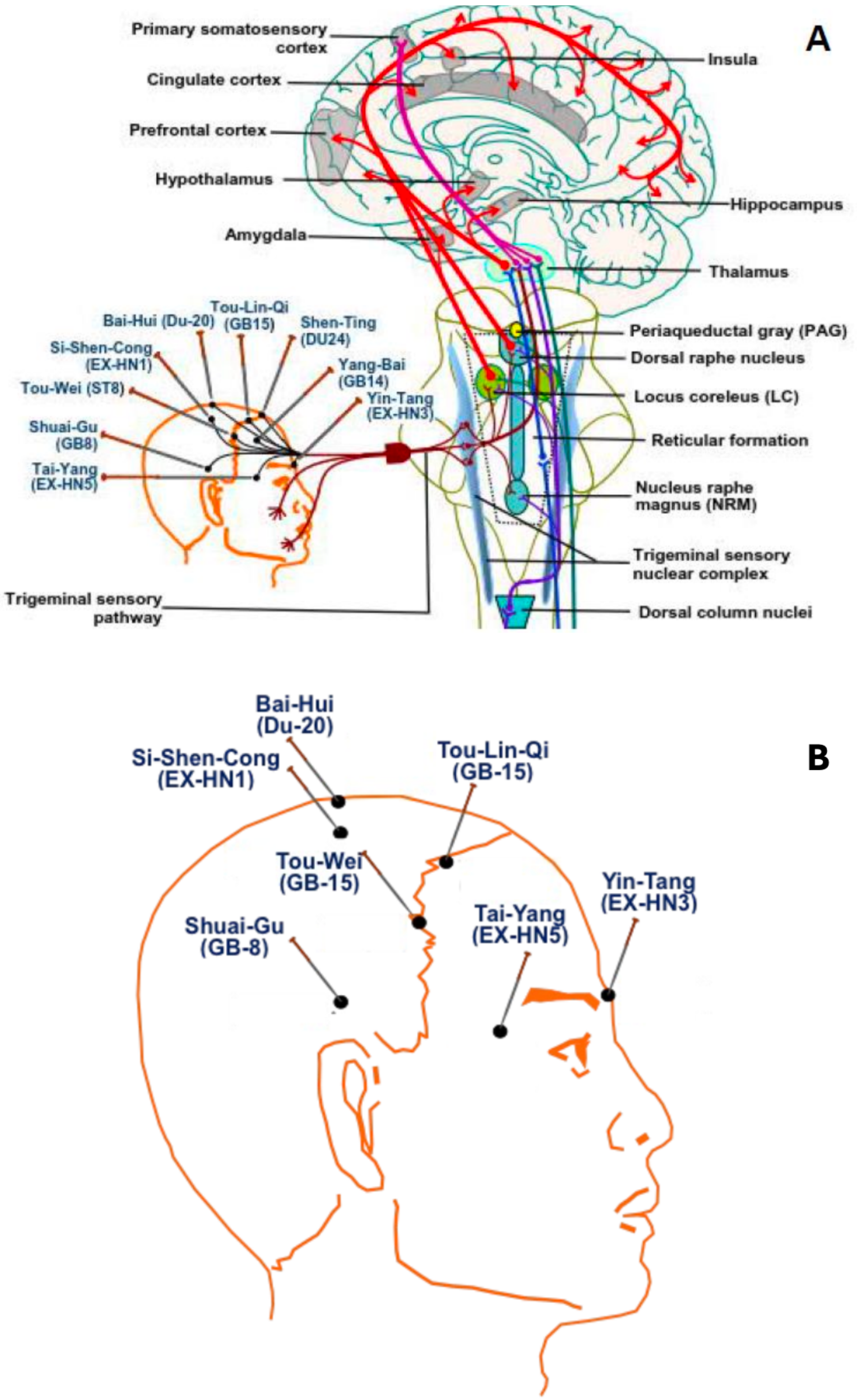

Fig. 3. Study flow chart. Abbreviations: T, timepoint; TEAS, transcutaneous electrical acupoint stimulation; DCEAS, dense cranial electroacupuncture stimulation.

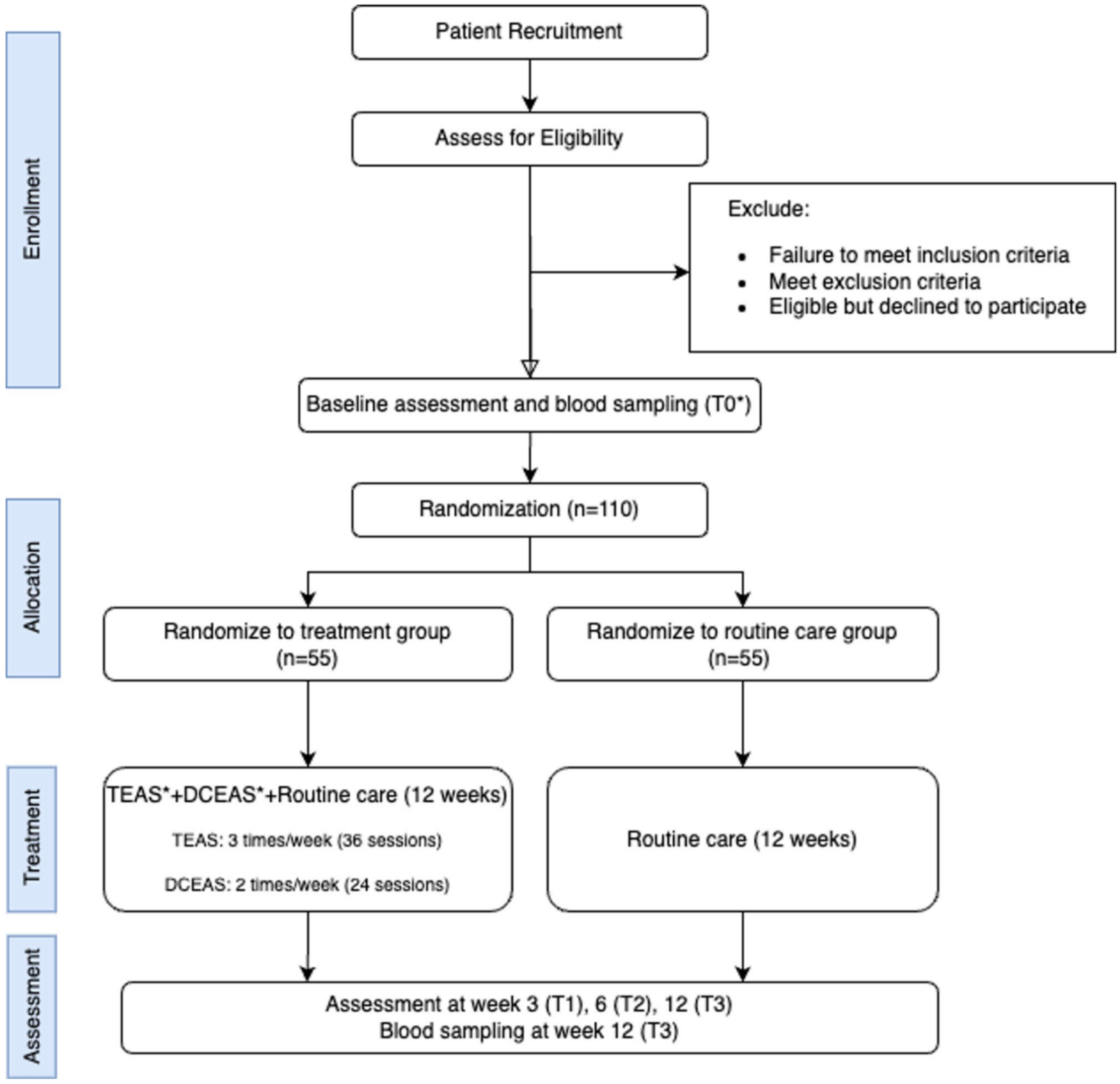

Fig. 4. SPIRIT schedule of enrolment, interventions, and assessments. Abbreviations: BDI-II, Beck depression inventory–II; AAS, abuse assessment screen; HAMD-17, 17-item Hamilton depression rating scale; PCL-C, PTSD check list-civilian version; PSS-10, 10-item perceived stress scale; ISI, insomnia severity index; SF-12, 12-item short form survey.

	STUDY PERIOD				
	**Enrollment**	**Allocation**	**Post-allocation**		
**Time point**		T0	T1	T2	T3
**Week**		0	3	6	12
ENROLLMENT:					
Eligibility screen	X				
Informed consent	X				
Demographics	X				
Allocation		X			
INTERVENTIONS (TEAS+DCEAS):					
Treatment group			⟵	⟶	⟶
Routine care group					
ASSESSMENT:					
BDI-II	X	X	X	X	X
AAS	X	X	X	X	X
HAMD-17		X	X	X	X
PCL-C		X	X	X	X
PSS-10		X	X	X	X
ISI		X	X	X	X
SF-12		X	X	X	X
Adverse Events		X	X	X	X
Blood Sampling		X			X
